# Biological Motion Primes the Animate/Inanimate Distinction in Infancy

**DOI:** 10.1371/journal.pone.0116910

**Published:** 2015-02-06

**Authors:** Diane Poulin-Dubois, Cristina Crivello, Kristyn Wright

**Affiliations:** Department of Psychology, Centre for Research in Human Development, Concordia University, Montréal, Québec, Canada; Eberhard Karls University of Tuebingen Medical School, GERMANY

## Abstract

Given that biological motion is both detected and preferred early in life, we tested the hypothesis that biological motion might be instrumental to infants’ differentiation of animate and inanimate categories. Infants were primed with either point-light displays of realistic biological motion, random motion, or schematic biological motion of an unfamiliar shape. After being habituated to these displays, 12-month-old infants categorized animals and vehicles as well as furniture and vehicles with the sequential touching task. The findings indicated that infants primed with point-light displays of realistic biological motion showed better categorization of animates than those exposed to random or schematic biological motion. These results suggest that human biological motion might be one of the motion cues that provide the building blocks for infants’ concept of animacy.

## Introduction

Evidence from various lines of research supports the assertion that infants form categories in a top-down manner, from most to least inclusive [1–5]. Infants are first able to form global or superordinate-level categories (e.g., animals vs. furniture) before being able to categorize narrower, less inclusive categories such as basic-level categories (e.g., cats vs. dogs; chairs vs. tables). In a seminal paper on the development of object categories in infancy, Mandler and Bauer [6] reported basic-level categorization in infants as young as 16 months of age using a sequential touching task, when the basic-level categories came from differing superordinate categories (e.g., dogs vs. cars are from the *animal* and *vehicle* category) but not when the contrasts were from the same superordinate category (e.g., cars vs. trucks are both from the *vehicle* category). Using the same sequential touching task, the authors found that 18-month-old infants were able to differentiate superordinate-level categories (animals vs. vehicles) but not basic-level categories of low contrast (e.g., dogs vs. horses) or moderate contrast (e.g., cars vs. motorcycles). By 30 months of age, infants were capable of discriminating low and moderate degrees of contrast at the basic-level [2]. In a more recent study, Bornstein and Arterberry [1] systematically examined categorization at four different levels of inclusiveness in 12- to 30-month-old infants. While categorization at a more inclusive level (e.g., superordinate-level ‘animals’ such as ducks, lions, pigs, and porpoises) was found to be above chance by 18 months of age, 12-month-old infants only showed a trend to categorize at the most inclusive level. This conflicts with evidence for the precocity of the animate-inanimate distinction when other tasks are used, such as the object examination procedure [7, 8]. Both 9- and 11-month-old infants categorize superordinate-level categories of animals and vehicles, while 7-month-old infants demonstrate a slightly lower level of performance. Recently, a study using event-related potentials with an oddball paradigm showed that 7-month-olds show a stronger novelty response to an oddball stimulus from a different superordinate level category (e.g., animal vs. furniture) than from the same category [9]. The main goal of the current study was to determine if 12-month-old infants are able to categorize animate and inanimate objects in the sequential touching task when provided with a priming stimulus common to animals, that is, biological motion.

While there is mounting evidence for the precocious acquisition of the animacy concept, much less is known about the perceptual cues infants use to acquire such an “abstract” concept. There are two broad classes of perceptual cues that could contribute to the identification of entities as animate or inanimate: featural cues (e.g., face, wheels) and dynamic cues (e.g., self-propulsion, movement upon contact). It has been hypothesized that infants form conceptual categories by extracting both static morphological and dynamic features of objects and use this information to determine an object’s category membership [10–12]. While it may be possible to categorize perceptually dissimilar objects using morphology alone (e.g. differentiation of animals and vehicles using object parts or general shape), the differentiation of perceptually similar items (e.g., birds and airplanes) likely requires more sophisticated knowledge of dynamic attributes [7]. Mandler [10, 13, 14] has hypothesized that it is the perception of motion characteristics that provides infants with conceptual knowledge about the “kinds of things” objects are. Specifically, she proposed that infants’ animate-inanimate conceptual categories are formed on the basis of conceptual primitives, which involve the movement of objects in space. These conceptual primitives may include whether objects start moving by themselves, whether they interact with other object*s*, and the kind of path they take. Similarly, Rakison and Poulin-Dubois [12] proposed that the foundation of the animate-inanimate distinction in infancy relies on the following five motion cues: a) *onset of motion* (self-propelled vs. caused motion), b) *type of causal role* (agent vs. recipient), c) *form of causal action* (action at a distance vs. action from contact), d) *pattern of interaction* (contingent vs. non-contingent), and e) *line of trajectory* (irregular vs. smooth).

The current research was designed to examine whether *manner of motion* (biological vs. non-biological), a basic animacy cue, facilitates animate-inanimate categorization in infancy. Biological motion refers to the characteristic, non-rigid patterns produced when humans and other animals move constrained by their skeletal structure. These biomechanical motion patterns are informative about an agent’s identity, emotional state, and activity, and adult observers are highly attuned to these features [15]. Biological motion processing is fast and automatic in adults and involves specialized cortical mechanisms, most notably the posterior superior temporal sulcus (pSTS, e.g., [16, 17]). Biological motion is typically studied by placing point-lights on the joints of the body, which is otherwise rendered invisible in the dark [18]. Point-light displays have been used to investigate the perception of biological motion across the lifespan. The ability to correctly recognize point-light biological motion of humans and animals is comparable to adults by 5 years of age [19]. In infancy, visual preference for biological motion has been demonstrated in newborns [20, 21] and 3-month-old infants [22, 23]. Further, 12-month-old infants extract social-cognitive cues, such as gaze following, from a point light display of a human walking [24]. Although 9-month-olds have been shown to categorize point-light displays of animals [25], the notion that biological motion perception is integral to infants’ differentiation of animate and inanimate concepts has received no empirical attention.

It has been suggested that the presentation of biological motion using point-light display confounds motion and form perception. That is, although no explicit shape information is provided, the body shape is implicit in the display and could be used when processing this type of stimulus [26]. Another stimulus that generates the perception of animacy is the schematic, non-rigid “caterpillar” motion [27]. Schematic presentations of biological motion, such as the Michotte “caterpillar,” depict a rectangular-shaped stimulus that moves by elongating from one side, then contracting on the opposite side. This stimulus has been shown to elicit the perception of goal-directedness in infants as young as 6 months of age [28] and is judged as ‘animal-like’ in children as young as 3 years of age [29]. Such judgments must be based on motion alone, as the rectangular shape of the stimulus is not animate in its morphology.

Given that biological motion is detected and preferred early in life, biological motion might be instrumental to infants’ differentiation of animate and inanimate concepts. One way to test whether this type of animate motion is a dynamic cue that infants use to facilitate the animate-inanimate distinction is by priming infants and observing improvement in their categorization performance. Priming involves the facilitation or activation of a concept by way of providing other conceptually related information [30]. In previous research, priming has been successfully used to increase social affiliation in very young children and to influence which level of inclusiveness infants focus on when categorizing objects [31, 32].

The overall objective of this study was to determine whether priming 12-month-old infants with biological motion improves their ability to categorize animate and inanimate objects when assessed with the sequential touching task. We investigated whether a point-light display of a human primes categorization of animals in 12-month-old infants. Previous research has shown that about 50% of 12-month-old infants categorize at the global level with a mean run length just at trend level [1]. Given that the human point-light display has been shown to also contain morphological cues [33], we view this prime as investigating the combined influence of motion and morphology. We also presented infants with a schematic biological motion prime, the Michotte stimulus, to provide a more stringent test of the potential effect of biological motion per se. We hypothesized that infants who were primed with human biological motion or schematic biological motion would categorize animals and vehicles better than infants who viewed random motion. In contrast, infants’ categorization of inanimate furniture-vehicle contrasts was not expected to be facilitated by these priming stimuli.

## Method

### Participants

A total of 106 infants were tested. Infants had no reported birth complications, as well as no visual or auditory impairment. Seventy-nine infants were included in the final analysis (*M age* = 12.36 months, *SD* =.42, age range: 11.69 to 13.40 months; 42 males) as the remaining 27 participants were excluded due to fussiness (*n* = 17), throwing toys (*n* = 8), and parental interference (*n* = 2). The experimental procedures were approved by the Human Research Ethics Committee of Concordia University, and all the parents involved were informed and consented (in written form) to let their child participate prior to data collection. All participants were free to withdraw from the experiment at any time.

### Materials

**Human Biological Motion Prime.** The human point-light walker video was composed of 11 point-light dots placed on all the major joints of the body. The walker moved rightward with no horizontal translation, 20 steps (10 gait cycles). In the creation of the human walker, eleven marker positions were used to capture the subject’s motion at all the major joints of the body. These markers convey important information about both the structure of the body (where the various joints and bones are located) and the dynamic movements of each part (e.g., the velocity of the arm swing vs. the stability of the trunk) [33]. The final video consisted of one 15-second trial, containing 30 complete gait cycles (0.5 seconds/cycle).

**Schematic Motion Prime.** The stimulus presented in this video was adapted from Michotte’s [27] schematic, non-rigid, caterpillar motion. A blue square (1.5 cm x 1.5 cm) expanded towards the right, while the left side remained still, forming the shape of a rectangle (1.5 cm x 3.5 cm). Next, the rectangle contracted wherein the right side remained still and the left side moved until the shape returned to its original form. The overall motion pattern of the schematic form was a rightward horizontal translation. This expansion-contraction motion was repeated six times for a trial duration of 15 seconds. At the beginning of each new trial, the square reappeared on the left side of the screen and moved rightward.

**Random Motion Control Prime.** The random motion control video used the same 11 point-light dots as the human biological motion display. Each dot was given a smooth line of trajectory and a fixed speed using VPixx© software [34]. None of the dots contained animate cues, such as the ability to change direction, change speed, or move contingently with other dots. The direction of the point-light dots was also controlled by having the same amount of dots moving to the right, left, up, or down.

### Procedure

Infants were randomly assigned to the human biological motion prime condition (*n* = 25), the schematic motion condition (n = 28), or the random motion control condition (*n* = 26). Infants viewed the video prime on a 61 cm x 36 cm screen placed at eye level, approximately 100 cm from the infant. Infants’ gaze was filmed and displayed on a computer monitor. Looking times were coded live using Habit 2000 software [35]. At the beginning of each trial, an attention getter (e.g., moving shape with sound) was used to orient infants’ gaze to the screen. This same stimulus also appeared when the infant looked away for more than two seconds. Each trial was 15 seconds in duration and infants were given a maximum of 14 trials to habituate. Infants were considered habituated once they reached the criterion, defined as three successive trials where the infants’ looking time was less than half of their looking time on the first three trials [36].

**Sequential Touching Task.** The sequential touching task is an implicit measure of infants’ ability to differentiate categories by measuring infants’ sequence of touches toward an array of toys from contrasting categories. Categorization is typically inferred if the infant touches objects from the same category in sequence before touching objects from the other category [37]. This procedure is considered appropriate for children between the ages of 13 and 30 months [38]. Infants were given a tray of eight toys containing four exemplars from two contrasting categories. The experimenter instructed infants by saying, “Look at all these toys. These toys are for you!” as a sweeping motion was made over the toys. Infants were given the opportunity to explore the toys without constraint for two minutes [39]. If infants dropped a toy on the ground, the experimenter simply placed the toy back on the tray.

Two sequential touching trials were administered in counterbalanced order: animal-vehicle and furniture-vehicle. The animal-vehicle tray was comprised of four animals (pig, lion, duck, dolphin) and four vehicles (boat, tractor, car, truck). The furniture-vehicle tray was comprised of four pieces of furniture (grandfather clock, desk, chair, TV stand) and four vehicles (train, sports car, bus, plane). During the testing session, participants were placed in a high chair and their parent sat behind them. Parents were advised to not direct their child’s attention to any toys (e.g., pointing or labeling). After the participants viewed the priming videos, the sequential touching task was administered.

**Coding and Reliability.** The sequences of touches to objects on both trays were coded for each participant. Thus, infants received separate scores for the animal-vehicle and furniture-vehicle trays. A touch was considered only if the infant coordinated touching using his/her hand, finger, or another object with eye gaze toward the object. Therefore, the following behaviors were not counted as a touch: if the infant accidentally touched an object without coordinated gaze or if the infant touched an object immediately after it was placed back on the tray (if dropped). Furthermore, to ensure that the infant was actively associating the objects touched in sequence, a delay between touches of 10 seconds or more was coded as a break in the run. Simultaneous touching of two objects from the same category was considered a single touch, while simultaneous touches of objects from different categories did not constitute a touch. Additionally, infants had to touch at minimum of one toy from each category, and throw fewer than three toys per categorization tray in order to be included.

A mean run length (MRL) for each tray of objects was calculated by dividing the total number of touches across categories by the total number of runs (i.e. touches to objects of the same category). The MRL for both trays was compared against chance (1.75). Chance is calculated using a formula that takes into account the total number of categories (2) and objects used (n) in the procedure [37]. A MRL of 1.75 or lower indicates that the infant touched the objects in a random order or in alternation; however, a MRL statistically above 1.75 is interpreted to indicate that infants performed successive touching. It has been argued that successive touching (above chance) reflects infants’ processing of within-category similarity. In contrast, alternating touching (below chance) reflects attention to between-category differences. Successive touching is considered a more advanced form of categorization that emerges later in infancy [40]. Since we predicted that priming would facilitate a mature form of categorization consisting of above chance performance (i.e. statistically greater than 1.75), one-tailed t-tests were used. An independent observer blind to the priming condition coded 25% of the sample to assess inter-rater reliability. A Pearson correlation was computed as *r* = 0.93 for the human biological motion condition, *r* =.88 for the schematic motion condition, and *r* =.93 for the random motion condition.

Although MRL analyses are informative to assess the categorical abilities of groups of individuals, this type of analysis tells us little about an individual child’s knowledge of categories, nor of the type of touching that took place. Therefore, an additional approach for analyzing children’s sequential touching was employed. This was to ascertain whether a child’s touches were primarily aimed toward either category or equally toward both categories. As outlined by Dixon, Price, Watkins, and Brink [41], children’s sequential touching was coded for “special” runs, which consist of touching a minimum of three different objects from the same category (either animal or vehicle) in succession. Based on these ‘special runs’, each participant was then classified as a noncategorizer, a single categorizer, or an exhaustive (dual) categorizer. Noncategorizers refer to those participants with no special runs in either category. Single categorizers refer to those participants with at least one special run in only one category (animal *or* vehicle). Finally, exhaustive categorizers refer to participants with at least one special run in both categories (animal *and* vehicle). The entire sequence of touches for each participant that contains the special runs was then entered into a Monte Carlo program (TouchStat 3.0) [41] to determine if they were likely to have occurred by chance. The program then simulated 10,000 random touch sequences in order to determine the frequency of occurrence of these special runs. Based on Mandler et al. [37], a probability lower than.10 (*p* <.10) signified that the participant’s run was unlikely to be due to chance alone. Based on the probability results, we determined whether each participant still qualified for their single categorizer or exhaustive categorizer status. The percentage of participants in each category was then calculated using the results of the Monte Carlo analyses.

## Results

The infants assigned to the three conditions did not differ in terms of demographic characteristics such as age or sex (age: *F*(2, 76) = 1.39, *p* = .26, partial η^2^ = .04; sex: χ^*2*^(2) = .98, *p* = .61, φ = .11). Skew and kurtosis values were examined for all dependent variables to ensure that the data were normally distributed. According to Kline [42], a skew of less than 3 and a kurtosis of less than 10 are acceptable, and thus the present data did not violate the assumption of normality. To test whether infants habituated to the priming videos, mean looking times during the first and last three trials of the habituation phase were compared. These two scores were entered into a 2 x 3 (Trial Block X Condition) mixed-design ANOVA. The analysis only revealed a significant main effect of Trial Block, *F*(1,76) = 374.66, *p* <.001, partial η^2^ = .83, wherein infants looked longer during the first block of trials (*M* = 9.22, *SD* = 2.63) than during the second block (*M* = 4.31, *SD* = 1.80). Thus, infants habituated to the video primes in all three conditions.

**Figure 1:**
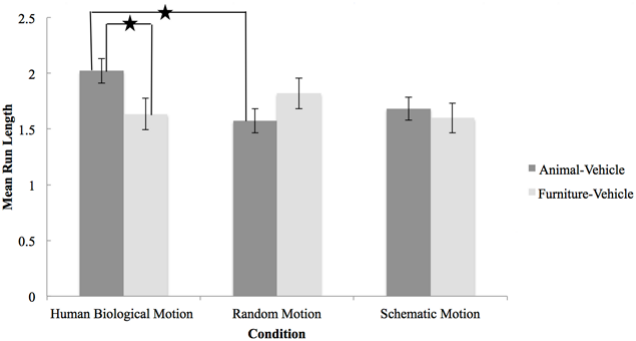
Mean Run Length as a Function of Priming Condition and Category. The Mean Run Length (MRL) was calculated for the Human Biological Motion, the Random Motion, and the Schematic Motion conditions across categorization of animal-vehicles and furniture-vehicles. A MRL of 1.75 refers to categorizing at chance level. In the present study, the human biological motion point-light display primed infants’ ability to categorize animals and vehicles, but not inanimate categories (e.g., furniture and vehicles). This priming effect was not found in the two other conditions.

To investigate whether priming infants with biological motion had an effect on categorization, a 2 (Category [animal-vehicle, furniture-vehicle]) x 3 (Condition [human biological motion, schematic motion, random motion]) mixed-design ANOVA was computed on mean run lengths. No main effect but a statistically significant Category x Condition interaction was found, *F*(2, 76) = 4.31, *p* = .02, partial η^2^ = .10. Pairwise comparisons (with Bonferroni corrections) revealed significant differences between categorization trials (animal-vehicle vs. furniture-vehicle) for the human biological motion condition, *M difference* =.39, *p* = .01. In contrast, mean differences in MRLs were not significant in the schematic motion condition (*M difference* = .09, *p* = .56) or in the random motion condition (*M difference* = -.25, *p* = .11). When comparing groups on categorization of furniture and vehicles, infants in the human biological motion condition (*M* = 1.63, *SD* = .49) had a MRL equivalent to infants in the schematic motion condition (*M* = 1.60, *SD* = .47; *M difference* = .035, *p* = 1.00) and in the random motion condition (*M* = 1.82, *SD* = 1.01; *M difference* = -.19, *p* = 1.00). In contrast, infants primed with human biological motion had a higher mean MRL for the animal-vehicle categorization (*M* = 2.02, *SD* = .75) than infants primed with the schematic motion (*M* = 1.68, *SD* = .48; *M difference* = .39, *p* = .09) and the random motion (*M* = 1.57, *SD* = .35; *M difference* = .45, *p* = .01) (see Fig. 1).

To compare the MRL of each category and condition to chance (MRL = 1.75) a one-sample t-test was used. Infants in the human biological motion condition performed above chance when categorizing animals and vehicles, *t*(24) = 1.87, *p* =.04, but not when categorizing furniture and vehicles, *t*(24) = -1.25, *p* =.22. The proportion of runs (23%) that included the dolphin was in line with the expected proportion based on four animals (25%). This indicates that biological motion primed the concept of animals, and not simply a body part such as legs. Infants in the schematic motion condition performed at chance when categorizing animals and vehicles, *t*(27) =-.73, *p* =.22, but below chance when categorizing furniture and vehicles, *t*(27) = -1.71, *p* =.05. Infants in the random motion condition performed below chance when categorizing animals and vehicles, *t*(25) = -2.54, *p* =.01, while the MRL for the furniture-vehicle trial did not differ from chance, *t*(25) =.42, *p* =.34.

**Monte Carlo analyses**. Children were each classified as categorizers or noncategorizers for each contrast in each condition using Monte Carlo simulation [43]. Table 1 shows the percentages of children in each condition classified as categorizers, collapsing across single and dual categorizers for the animal and vehicle contrasts as well as the furniture and vehicle contrasts. In terms of category pairs, about one-half of children categorized the animals vs. vehicles after being primed with the human point-light walker (48%). In contrast, much fewer children categorized animals vs. vehicles following exposure to schematic motion (28%) or random motion (11%). As expected, categorization of artifacts was not facilitated by human point-light biological motion (28%), schematic biological motion (27%), or random motion (14%) priming.

**Table 1:** Mean percentage of categorizers across condition and type of category.

	**Priming Condition**		
**Category trial**	**Human Biological Motion**	**Random Motion**	**Schematic Motion**
Animal-vehicle	48%	11.5%	28.6%
Furniture-vehicle	28%	26.90%	14.3%

## Discussion

The purpose of this experiment was to investigate if exposure to biological motion primes infants’ categorization of animals and vehicles using a sequential touching task. The results provide support for the hypothesis that a human biological motion point-light display primes infants’ ability to categorize animals and vehicles. Further, this effect was not observed when infants were primed with schematic biological motion, or random motion. Importantly, the priming effect was specific to the animal-vehicle categorization task and did not influence infants’ performance on the task requiring infants to differentiate two inanimate categories (e.g., furniture and vehicles). Additional support for our hypothesis is demonstrated by the fact that infants in the human biological motion condition categorized above chance (MRL > 1.75) on the animal-vehicle task, but not on the furniture-vehicle task. None of the mean run lengths in the other two conditions was above chance. This is an extraordinary finding for two reasons: 1) in a point-light display, the movement of the body is reduced to the motion of dots that represent the key joints, and 2) the movement of a *human* body facilitated the categorization of animals, including mammals and birds. These results can be interpreted as support for a precocious association of biological motion with animacy by 12 months of age, as long as the structure of the human body is implicit in the point-light display.

In contrast to previous research on categorization of animals vs. artifacts without priming, 12-month-old infants performed at a level above chance when tested with a sequential touching task [1]. The current results also demonstrated that biological motion alone is not sufficient to improve infants’ animal–vehicle categorization abilities. Infants in the schematic biological motion condition (‘Michotte’ stimulus) did not perform significantly better on the sequential touching task than those exposed to random motion. However, the schematic motion differed from the biological motion prime in many ways, including the absence of human locomotion. Future studies contrasting intact biological motion with other motion stimuli (e,g., scrambled motion) will be needed in order to clarify whether animate motion needs to contain form cues to prime the concept of animacy in a categorization task. Nonetheless, this finding contrasts with recent research showing that infants as young as 6 months of age attribute goals to schematic animal motion, like the moving square that we used [28], and that schematic biological motion cues might signal agency before animacy. However, associating goal-directedness to schematic biological motion does not entail, as the authors suggest, that the moving shape is perceived as animate. Similarly, goal attribution has been linked to a self-propelled box in 5-month-olds [44]. These findings do not provide information about whether infants perceive these objects as animate, only that they are able to associate motion cues considered to be conceptual primitives for the development of the animacy concept [14]. Furthermore, the absence of a priming effect for the schematic motion condition suggests that this type of motion is not identified as animal-like by infants, as it is by preschoolers and adults [29].

The research presented here contributes to understanding early conceptual development in many ways. First, because infants were primed with human biological motion and were tested on their ability to differentiate non-human animals (mammals and fish) from vehicles, these results provide evidence for the notion that infants as young as 12 months of age possess an implicit concept of animates, which includes both humans and animals. To date, only a handful of studies have addressed this issue. Poulin-Dubois, Frenkiel-Fishman, Nayer, and Johnson [45] reported that infants as young as 16 months of age extend motion and sensory properties modeled on people to animal exemplars (i.e., within the animate category). More recently, Rostad et al. [4] reported that even 14-month-olds can group animals and people together with a sequential touching procedure. We provide evidence that realistic point-light biological motion is recognized as animate by infants as young as 12 months. To date, research using point-light displays with infants have reported sensitivity to biological motion and recognition of the solidity of the human body [46]. Our data suggest that such displays also activate stored knowledge about animate beings broadly defined.

The second main contribution of the present study is to identify biological motion as an animacy cue that could assist infants in categorizing animals as different from vehicles. In addition to salient perceptual features (such as faces) that help infants identify some entities as an animal, it has been proposed that the animacy concept is derived from salient spatial information, especially movement in space [13]. Animals are self-propelled entities that interact with other objects from a distance, whereas inanimates do not move unless they are contacted by something else and they do not interact with other objects from a distance. Some research has investigated whether infants expect animals to move according to animate motion properties. For example, self-propelled motion has been found to be associated with human hands and animals by 7 months of age [47–50]. Infants expect an inert inanimate object to move only when contacted by another moving entity; however, these expectations are suspended for a person or other agent capable of self-generated motion [51–56]. To date, no research has examined if manner of motion (mechanical vs. biological) is also associated with the global categories of animals and vehicles. The findings of the present study support the hypothesis that biological motion is an important building block in the acquisition of the concept of animacy. Since even newborns preferentially attend to biological motion, it is likely the case that this ability facilitates categorization and not the reverse as some form of global categorization has been documented only a few months later with the familiarization-novelty procedure. Unlike self-propulsion, which appears to apply equally to animals and vehicles without knowledge about human agency in vehicle motion, biological motion might be uniquely associated with animacy from birth.

The priming paradigm developed here to assess whether infants associate biological motion with animals offers new ways to study the foundations for the development of early categorization. First, it could be easily adapted to test younger infants by pairing it with categorization tasks appropriate for infants younger than 12 months, such as the object examination procedure. Second, rigid, mechanical motion could be substituted for biological motion as the priming stimulus in order to determine if inanimates are associated with this manner of motion. One would expect that categorization of vehicles would be facilitated by exposure to, for example, a car rolling. Future research may also investigate whether priming with schematic biological motion will boost older infants’ concept of animacy. Although infants might show hard-wired sensitivity to the minimal information that the Michotte [27] caterpillar-like motion pattern provides, learning that this two-anchor crawling might have commonalities with 4-legged animals’ motion might require additional experience. Biological motion perception is remarkably robust and has immense evolutionary and social importance [19, 57]. The detection of biological motion is a fundamental perceptual process that is part of an early developing and evolutionarily-endowed mechanism shared across species. This ability to recognize and preferentially attend to the motion of biological entities, even when presented in its most rudimentary form, has been hypothesized to underlie developing social cognition (e.g., [24, 58]. Further studies should aim at expanding our knowledge of its role in the emergence and development of infants’ animate/inanimate distinction.
